# Lactonase Activity, Status, and Genetic Variations of Paraoxonase 1 in Women with Gestational Diabetes Mellitus

**DOI:** 10.1155/2020/3483427

**Published:** 2020-02-03

**Authors:** Mi Zhou, Xing-Hui Liu, Qing-Qing Liu, Meng Chen, Huai Bai, Lin-Bo Guan, Ping Fan

**Affiliations:** ^1^Department of Obstetrics and Gynecology, West China Second University Hospital, Sichuan University, Chengdu, 610041 Sichuan, China; ^2^Laboratory of Genetic Disease and Perinatal Medicine, Key Laboratory of Birth Defects and Related Diseases of Women and Children, Ministry of Education, West China Second University Hospital, Sichuan University, Chengdu, 610041 Sichuan, China

## Abstract

**Background:**

Paraoxonase 1 (PON1) is a calcium-dependent multifunctional enzyme that binds to high-density lipoproteins. The physiological function of PON1 is related to its lactonase activity. However, this activity has not been analyzed in women with gestational diabetes mellitus (GDM). The present study investigated the lactonase activities and status of PON1 and their association with *PON1* genetic variants and oxidative stress indices in Chinese women with GDM.

**Methods:**

This is a case-control study of 347 women with GDM and 288 women with uncomplicated pregnancies. PON1 levels and lactonase activities were analyzed using 7-O-diethylphosphoryl-3-cyano-4-methyl-7-hydroxycoumarin (DEPCyMC) and 5-thiobutyl butyrolactone (TBBL), respectively. A normalized lactonase activity (NLA) was estimated based on the ratio of TBBLase to DEPCyMCase activity. Serum malondialdehyde (MDA), total oxidant status (TOS), total antioxidant capacity (TAC) levels, and *PON1* genetic variants and oxidative stress indices in Chinese women with GDM.

**Results:**

PON1 lactonase activity and levels of TOS, TAC, and MDA were higher in the GDM women compared with the control women. The *PON1 -108C→T* genetic variation decreased the levels and lactonase activities of PON1 in a genotype-dependent manner in the patient and control groups. GDM patients with the *PON1 -108TT* genotype displayed lower NLA than those with the *-108CC* or *-108CT* genotype. GDM patients with the *RR* genotype of *PON1 192Q/R* polymorphism had significantly lower PON1 lactonase activities and NLA and tended to have decreased PON1 levels compared with those with the *QQ* or *QR* genotype. Multivariable regression analysis revealed that the *PON1 -108C/T* or *192Q/R* variations, apolipoprotein (apo) A1, apoB, TAC, MDA, or age was significant predictors of the levels, lactonase activities, or NLA of PON1.

**Conclusions:**

The lactonase activities of PON1 are increased in women with GDM. *PON1* genetic variants, increased oxidative stress, and abnormalities in lipoproteins may be associated with these changes.*PON1* genetic variants and oxidative stress indices in Chinese women with GDM.

## 1. Introduction

Gestational diabetes mellitus (GDM) is one of the most common metabolic disorders of pregnancy and is defined as any degree of carbohydrate intolerance that is first recognized during pregnancy [1]. The prevalence of GDM varies among different racial or ethnic groups [1, 2]. The prevalence of GDM is reported to be 14.8% in pregnant Chinese women [2]. With the increased prevalence of obesity, sedentary lifestyles, and older pregnant women, the prevalence of GDM has increased globally in recent years [1, 2]. GDM can seriously harm both the mother and fetus during pregnancy, including the increased risks of preeclampsia and macrosomia [1]. GDM is also associated with increased risk of metabolic-related diseases, such as type 2 diabetes mellitus, metabolic syndrome, and cardiovascular diseases in future life [1, 3]. GDM might be related to genetic variants [4], increased oxidative stress [5], dyslipidemia [6], and chronic inflammation [7].

Paraoxonase 1 (PON1) is a calcium-dependent multifunctional enzyme with arylesterase (AREase), paraoxonase (POase), Hcy-thiolactonase (HTase), and lactonase activities [8–10]. PON1 is mainly synthesized by the liver and combines with high-density lipoprotein (HDL) in the blood circulation [9, 10]. The enzyme hydrolyzes several nerve agents and organophosphorus insecticides to protect against xenobiotic toxicity and also attenuates low-density lipoprotein (LDL) oxidation, reduces macrophage foam cell formation, and degrades homocysteine thiolactone. Thus, PON1 plays a pivotal role in antioxidative, anti-inflammatory, and antiatherogenic responses [8, 10]. It has been suggested that the physiological functions of PON1 are related to its lactonase and HTase activities [8, 10] and that the POase and AREase activities of PON1 are promiscuous activities [10, 11]. Several studies have also proven that the active sites for the lactonase and promiscuous activities of PON1 are different [10, 12, 13] and that PON1 is able to hydrolyze lipid peroxides due to its lactonase activity [10, 13]. Thus, compared with the POase and AREase tests, the lactonase test of PON1 can better reflect the antioxidative and antiatherogenic properties of PON1 [10, 12, 14].

Some *PON1* genetic variations have been reported to influence the enzyme concentration and specific activity of PON1 [10, 15]. The *−108C/T* variant of the *PON1* gene influences PON1 expression and the *192Q/R* polymorphism affects PON1 activities [9, 10, 16]. The PON1 R isoform hydrolyzes lipid peroxides in vitro less efficiently than does the Q isoform [9]. The *192Q/R* polymorphism has been reportedly associated with coronary heart disease [9, 10], polycystic ovary syndrome [17], and GDM [18, 19].

POase and/or AREase activities of PON1 are decreased in GDM [20, 21]. However, the lactonase activity and status of PON1 have not yet been analyzed in women with GDM. It also remains unknown whether there are any relationships between the *192Q/R* and *-108C/T* polymorphisms of the *PON1* gene and GDM in Chinese women. In the present study, we investigated the lactonase activity and status of PON1 and their association with the *−108C/T* and *192Q/R* variants of the *PON1* gene and oxidative stress indexes in Chinese women with GDM.

## 2. Materials and Methods

### 2.1. Study Subjects

This is a case-control study of 347 women with GDM and 288 women with uncomplicated pregnancies. The subjects were recruited from the Department of Obstetrics and Gynecology of West China Second University Hospital between 2013 and 2018. The study was carried out in accordance with the Declaration of Helsinki. Written informed consent was obtained from all of the participants. This study was approved by the Institutional Review Board of the West China Second University Hospital, Sichuan University (No. 2017-033).

All pregnant women at 24–28 gestational weeks underwent a routine 75 g oral glucose tolerance test. The diagnosis of GDM was based on the guidelines of the International Association of Diabetes Pregnancy Study Groups (IADPSG), which specifies one or more of the following findings: fasting glucose ≥ 5.1 mmol/L, 1 h glucose ≥ 10.0 mmol/L, or 2 h glucose ≥ 8.5 mmol/L [22]. The control women with uncomplicated pregnancies were recruited from the aforementioned department of the hospital during the same period. The inclusion criteria of the subjects were elective caesarean section and singleton pregnancy.

The exclusion criteria for the subjects were diabetes mellitus before pregnancy; chronic hypertension; any other pregnancy complications including preeclampsia and intrahepatic cholestasis of pregnancy; autoimmune, cardiac, renal, and hepatic diseases; and emergency caesarean delivery or multiple pregnancy.

Clinical and anthropometrical variables including the systolic blood pressure (SBP), diastolic blood pressures (DBP), and body mass index (BMI, kg/m^2^) were measured or assessed.

Blood samples of the study subjects were obtained in the morning of the elective caesarean section after an overnight fast. The blood samples were kept on ice and centrifuged at 1500 × g for 15 min at 4°C. The plasma and serum aliquots were stored at -80°C for later analysis. The collection and separation of samples were completed within 4 h.

### 2.2. Analysis of PON1 Activity, Metabolic, and Oxidative Stress Markers

The lactonase activity and levels of PON1 were determined using 5-thiobutyl butyrolactone (TBBL) and 7-O-diethylphosphoryl-3-cyano-4-methyl-7-hydroxycoumarin (DEPCyMC) as a substrate, respectively, based on the measurement described in our earlier study [16]. A normalized lactonase activity (NLA) = TBBL activity (U/mL) × 1000/DEPCyMC activity (mU/mL) [14, 16].

Serum total antioxidant capacity (TAC) was measured by the semiautomatic microplate colorimetric method using Trolox as a standard as previously described [23], with some modifications. Briefly, 8 *μ*L of serum sample or different concentrations of Trolox calibrator (0.125-2.5 mmol/L) were added to wells of a 96-well, polystyrene SpectraPlate (PerkinElmer, Inc.). Next, 200 *μ*L of Reagent 1 (0.4 mol/L acetate buffer solution, pH 5.8) was added to each well and mixed. The plate was placed in the Varioskan Flash Multimode Microplate Spectrophotometer (Thermo Fisher Scientific), and the first absorbance reading was taken at a wavelength of 420 nm as the blank sample. Then, 20 *μ*L of Reagent 2 (30 mmol/L acetate buffer solution, 3.2 mmol/L H_2_O_2_, 10 mmol/L 2,2′-azino-bis(3-ethylbenzothiazoline-6-sulfonic acid) (ABTS), pH 3.6) was mixed thoroughly and incubated at 28°C for 5 min. The second absorbance reading was taken at a wavelength of 420 nm. The concentration of TAC in the serum samples was obtained by computerized data reduction of the absorbance for the calibrators versus the concentration using a linearity model. The results are expressed in terms of millimolar Trolox equivalent per liter (mmol Trolox Equiv./L). The samples were plated in duplicate, and a pooled serum sample from the healthy volunteers was included on each plate as a quality control.

Serum total cholesterol (TC), HDL-cholesterol (HDL-C), LDL-cholesterol (LDL-C), triglyceride (TG), apolipoprotein (apo)A1, apoB, total oxidant status (TOS) and malondialdehyde (MDA) levels, plasma insulin, and glucose concentrations, as well as the homeostatic model assessment of insulin resistance (HOMA-IR), were measured or assessed as previously described [16, 24]. The atherogenic index (AI) was calculated using the following equation: AI = (TC‐(HDL − C))/(HDL‐C) [17]. The oxidative stress index (OSI) was expressed as the ratio of TOS to TAC.

The intra- and interassay coefficients of variation for all of the measurements were less than 5% and 10%, respectively.

### 2.3. Genotype Analysis

The genomic DNA was isolated from the peripheral blood leukocytes of the subjects and *PON1 192Q/R*, and *-108C/T* genotypes were assessed by PCR amplification and restriction analysis as previously described [16, 17, 25] with some modification. A total volume of 25 *μ*L containing 2.5 *μ*L of 10× PCR buffer, 1.5 mM MgCl_2_, 200 *μ*M of each dNTP, and 0.75 U of Taq polymerase (Thermo Fisher Scientific), 2.0 *μ*L of genomic DNA template (approximately 30-80 ng), 0.30 *μ*M of each primer: forward 5′-TATTGTTGCTGTGGGACCTGAG-3′ and reverse 5′-CACGCTAAACCCAAATACATCTC-3′ for the *192Q/R* genotype [17], and 0.20 *μ*M of each primer: forward 5′-GACCGCAAGCCACGCCTTCTGTGCACC-3′ and reverse 5′-TATATTTAATTGCAGCCGCAGCCCTGCTGGGGCAGCGCCGATTGGCCCGCCGC-3′for the *-108C/T* genotype [25] were used. The PCR was performed with predenaturation at 95°C for 3 min followed by 32 cycles of 45 sec at 95°C, 45 sec at 61°C, and 60 sec at 72°C for the *192Q/R* genotype and 29 cycles of 1 min at 95°C, 1 min at 70°C, and 1 min at 72°C for the *-108C/T* genotype and ending with a single 7 min extension step at 72°C. Five microliters of the *192Q/R* (99 bp) or *-108C/T* (119 bp) PCR products was digested with 1 U of AlwI (New England Biolabs, Inc.) or 4 U of BstuI (Thermo Fisher Scientific) in a 10 *μ*L reaction volume for 1-16 h at 37°C. The digestion resulted in 66 and 33 bp fragments for the *192R* allele and a nondigested 99 bp fragment for the *192Q* allele, 67 and 52 bp fragments for the *-108C* allele and a nondigested 119 bp fragment for the *-108* T allele. The products were analyzed by 8.0% PAGE gel or 3.5% agarose gel electrophoresis. The resolved DNA fragments were visualized by staining with GelRed or Genecolour fluorescent dye, respectively.

### 2.4. Statistical Analyses

For the variables with normal distribution, the data are expressed as the mean ± standard deviation (SD), and differences in variables between the GDM and control groups were evaluated with an independent sample *t*-test. For variables with asymmetric distribution, the data are expressed as the median (lower limit of IQR-upper limit of IQR). If the variable showed normal distribution after logarithmic transformation, the difference in variables between the two groups was evaluated by the independent sample *t*-test. If not, the difference was evaluated by the Mann–Whitney *U* nonparametric test. The analysis of covariance (ANCOVA) was used to analyze the differences of parameters between/among the groups after correcting for differences in age and BMI at delivery. The chi-squared (x^2^) test was used to assess deviations of the genotype distribution from the Hardy–Weinberg equilibrium and to determine the allele or genotype frequencies between the patients and controls. The influence of *PON1* genotypes on the oxidative stress parameters and the activities and levels of PON1 were calculated with an analysis of variance (ANOVA) or ANCOVA. The effects of other parameters including the *-*108*C/T* and 192*Q/R* genotypes of PON1; GDM status (GDM = 1, control = 0); age, weight gain during pregnancy, delivery BMI, and gestation age; fasting glucose and insulin levels; and HDL-C, LDL-C, TG, apoA1, apoB, MDA, TOS, and TAC on the lactonase activity, level, or NLA of PON1 were evaluated by the multivariate stepwise regression analyses. Pearson's correlation and the multivariate stepwise regression analyses were used to test the associations of the levels, activities, and genetic variants of PON1 with characteristics of the neonates. A value of *P* < 0.05 was considered statistically significant. All of the statistical analyses were conducted using the Statistical Program for Social Sciences (SPSS) 21.0.

## 3. Results

### 3.1. Clinical and Biochemical Characteristics of the Women with and without GDM

The women with GDM were older, had higher prepregnancy BMI and SBP, and lower weight gain during pregnancy compared with the control women. Of the 347 women with GDM, 49 women required insulin treatment and 298 women had dietary and exercise-based treatment only (Table 1).

The women with GDM also had significantly higher fasting glucose and insulin concentrations, HOMA-IR, TG, TG/HDL-C ratios, TOS, TAC and MDA levels, and PON1 lactonase activities and tended to have increased PON1 levels compared with the control women after adjusting for the difference in age and BMI at delivery (Table 2).

### 3.2. Distribution of PON1 -108C/T and 192Q/R Genotypes and Alleles

The genotypic distributions of the *PON1 -108C/T* and *192Q/R* polymorphisms were in Hardy–Weinberg equilibrium in the women with and without GDM (all *P* > 0.05). No significant differences were observed in the frequencies of the *PON1 -108C/T* and *192Q/R* genotypes and alleles between the GDM and control groups (Table 3).

### 3.3. Lactonase Activity, Level, and NLA of PON1 and Oxidative Stress Indexes according to PON1 Genotypes in Women with and without GDM

As exhibited in Table 4, the *PON1 -108C→T* variants decreased PON1 levels and PON1 lactonase activities in a genotype-dependent manner (*CC > CT > TT*) in the women with and without GDM. The NLA was significantly lower in the *PON1 -108TT* genotype than in the *-108CC* or *-108CT* genotype in the women with GDM, and the TOS levels tended to have increased in the *-108TT* genotype than in the *-108CT* genotype in the control group (*P* = 0.061).

The GDM women with the *RR* genotype of *PON1 192Q/R* polymorphism had significantly lower PON1 lactonase activities and NLA (*P* < 0.05) and tended to have decreased PON1 levels (*P* < 0.095) compared with those with the *QQ* or *QR* genotype. The GDM women with the *QR* genotype tended to have decreased NLA compared with the GDM women with the *QQ* genotype (*P* = 0.099). The control women with the *R* allele (*QR* or *RR* genotype) had significantly lower PON1 lactonase activities and NLA, and those with the *QR* genotype had also lower TAC and MDA levels compared with the control women with the *QQ* genotype. The control women with the *RR* genotype had increased MDA levels and tended to have increased TOS and TAC levels (*P* < 0.065) and have decreased the lactonase activities and NLA of PON1 (*P* < 0.085) compared with the control women with the *QR* genotype (Table 4).

### 3.4. Association of Important Independent Variables with Lactonase Activity, Level, and NLA of PON1 in Women with and without GDM

Multivariate stepwise regression analysis revealed that the *PON1 -108C/T* polymorphism, age, apoA1, apoB, and TAC were significant predictors of the PON1 lactonase activities. The *PON1 -108C/T* and *192Q/R* variants, age, apoA1, apoB, and MDA were significant predictors of the PON1 levels, and the *PON1 192Q/R* variant, apoB, TAC, and MDA were significant predictors of the NLA in the women with and without GDM (Table 5).

### 3.5. Association of Lactonase Activities, Levels, and Genetic Variants of PON1 with Characteristics of Neonates in the Women with and without GDM

Bivariate analysis in women with GDM showed that the neonatal birth height correlated negatively with the levels and lactonase activities of PON1 (*r* = ‐0.189 and -0.147, respectively; all *P* < 0.01). The neonatal birth weight also correlated negatively with the levels and lactonase activities of PON1 (*r* = ‐0.155 and -0.128, respectively; all *P* < 0.05).

Multivariate stepwise regression analysis including the lactonase activities, levels, NLA, and *-108C/T* and *192Q/R* genotypes of *PON1* as independents showed that the PON1 levels were significant predictors of the neonatal birth height and weight in the women with and without GDM (Table 6).

## 4. Discussion

This study provides the first evidence that the lactonase activities and levels of PON1 are increased in women with GDM, indicating that a compensatory stimulation of PON1 lactonase activities and an increase of PON1 expression may be present in women with GDM. Furthermore, we established that the *-108C/T* and/or *192Q/R* variants of the *PON1* gene, increased oxidative stress, and abnormalities in the serum apoA1 and/or apoB levels may be related to these changes. In addition, we also found that the PON1 levels were significant predictors of the neonatal birth height and weight. However, our results did not document that the *PON1* gene *-108C/T* and *192Q/R* polymorphisms were associated with the risk of GDM in Chinese women.

Several studies reported reduced POase and/or AREase activities or levels of PON1 in women with GDM [20, 21]. However, different from previous studies, we found that, compared with the control groups, women with GDM displayed higher or relatively high levels and lactonase activities of PON1. These results are similar with our previous research demonstrating elevated levels and lactonase activities of PON1 in patients with polycystic ovary syndrome [16]. In addition, we confirmed that age was inversely associated with the levels and lactonase activities of PON1, which were consistent with the previous studies [26].

PON1 has a role as an antioxidant that involves the hydrolysis of lipid peroxides, which depends on its lactonase activity [9–11]. Impairing the lactonase activity of PON1 via mutations of the catalytic histidine dyad (H134Q and H115Q) weakens the ability of PON1 to prevent LDL oxidation and stimulate macrophage cholesterol efflux. These facts demonstrate that the antiatherogenic functions of PON1 may be mediated by its lactonase activity [13, 27]. TBBL is a synthetic chromogenic lactone that resembles the most natural PON1 lactone substrates. TBBL can specifically evaluate PON1 lactonase activity [16, 28]. The DEPCyMCase activities of PON1 have been shown to be a good marker of PON1 protein levels because they are not influenced by the degree of catalytic stimulation by HDL and can provide information similar to direct PON1 quantification by ELISA [14]. The AREase activity of PON1 is also regarded as a classical surrogate marker of PON1 concentration. However, it undergoes an approximately twofold higher stimulation upon HDL binding. Thus, the AREase activity may not be a good marker to evaluate the PON1 level [29]. The NLA of PON1, which is the ratio of TBBL : DEPCyMC activity, may reflect the level of PON1 lactonase catalytic stimulation by HDL [14, 30]. Thus, NLA can estimate the status of PON1 very well.

Women with GDM display increased lipid peroxides, 8-isoprostane, and MDA levels and decreased TAC and levels/activities of antioxidative enzymes that include superoxide dismutase, catalase, and glutathione peroxidase when compared with the corresponding control groups [5, 31]. In the present study, compared with the control women, the women with GDM displayed higher serum TOS, TAC, and MDA levels. Multivariable regression analysis in women with and without GDM revealed positive correlations of TAC with the lactonase activities and NLA of PON1, positive correlation of MDA levels with the levels of PON1, and negative correlation of MDA levels with the NLA of PON1. These findings suggest that the elevated lactonase activities and levels of PON1 and TAC might compensate for increased oxidative stress in women with GDM. On the other hand, the hyperglycemic condition has been correlated with accelerated generation of reactive oxygen species and protein glycation [32]. Glycation and/or oxidation of PON1 in HDL may lead to dissociation of PON1 from the HDL and inactivation and consumption of PON1 [33, 34]. This may explain the decreased serum PON1 activity and/or levels that are associated with increased PON1 protein expression in some diseases [35, 36]. In addition, we also found that the levels and lactonase activities of PON1 correlated negatively with the neonatal birth height and weight in women with GDM. However, the basis of this relationship remains unclear and requires further study. The data indicate that PON1 plays an important role in preventing oxidative stress and that the absolute or relative lack of PON1 lactonase activity may contribute to the pathogenesis of GDM.

Plasma lipoproteins and apolipoproteins have a significant effect on the activities of PON1 [37–39]. apoA1 or apoE combined with PON1 in HDL can markedly enhance the stability of PON1 and stimulate the lactonase activities of PON1 [37, 38]. The dissociation of PON1 from HDL to the lipoprotein-deficient serum fraction is accompanied by a loss of PON1 lactonase activities and antiatherogenic properties [40]. The proatherogenic small and dense LDLs have been associated with increased PON1 activity [39]. An increased TG/HDL-C ratio has been implicated as a sensitive and specific predictor of small and dense LDLs and insulin resistance [41]. In the present study, women with GDM had increased TG/HDL-C ratios, suggesting that there may be higher levels of small and dense LDLs in these patients. Multivariable regression analysis showed that serum apoA1 and/or apoB levels are significant predictors of the levels, lactonase activities, and NLA of PON1 in women with and without GDM. These findings suggest that the changes in the HDL composition or/and the presence of proatherogenic lipidemia might be related to a complementary stimulation of PON1 lactonase activity in women with GDM.

Several polymorphisms of the *PON1* gene may markedly influence protein expression and/or activities of PON1 and account for more than 60% of the interindividual variation in enzyme concentration and activity [9, 16, 37]. Promoter region *-108 C→T* variants of the *PON1* gene, a potential binding site for the transcription factor Sp1, decreases the PON1 expression [25, 42]. This decreases serum PON1 level and consequently serum PON activity [16, 43]. Presently, the *−108C/T* polymorphism accounted for 22.8% of the observed variability in PON1 expression levels, which was much greater than that attributable to the other PON1 polymorphisms [25]. The *192Q/R* polymorphism of the *PON1* gene significantly affects the PON1 activities in a substrate-dependent manner [9, 44]. It has been reported that the R isoform of PON1 hydrolyzes paraoxon (POase activity) more efficiently in vitro [15, 44], whereas the Q isoform hydrolyzes diazoxon, soman, sarin [44], and lipid peroxides [9, 10] more rapidly. However, data concerning the relationship between 192 isoforms and the rates of phenyl acetate hydrolysis (AREase activity) is contradictory [15, 25, 44]. Similar to previous findings, in the present study, *PON1 -108C→T* variants decreased the levels and lactonase activities of PON1 in a genotype-dependent manner (*CC > CT > TT*). *PON1 192Q→R* variants, especially *RR* homozygotes, and decreased the lactonase activities and NLA of PON1 in the women with and without GDM. We also showed that the *PON1 -108C→T* genetic variants were negatively correlated with the levels and lactonase activities of PON1 in the women with and without GDM. The *192Q→R* genetic variant was a crucial predictor of the levels and NLA of PON1 in the women with and without GDM in the multivariate regression models. The collective results demonstrate that the *-108 C→T* and *192Q→R* variants of the *PON1* gene significantly affect the lactonase activities and status of PON1 and decrease the levels and/or lactonase activities of PON1.

The *R* allele and the *192RR* genotype are associated with increased predisposition to GDM in Saudi or Greek women [18, 19]. However, in the present study, no significant differences were observed in the frequencies of the *PON1 -108C/T* and *192Q/R* genotypes and alleles between Chinese women with and without GDM. This inconsistency may be in part attributed to the various ethnic backgrounds among the different populations [9, 17, 25].

In addition to oxidative stress, genetic variations, and abnormal lipoprotein metabolism, other factors including dietary habits, consumption of antioxidants, and physical exercise may also affect PON1 expression and the levels and activities of PON1 in the circulation [45]. Further analyses of these factors may help provide clues to the mechanisms responsible for changes of the PON1 levels and lactonase activities in women with GDM. In addition, for an association study between *PON1* genotypes and the risk of GDM, the present sample sizes (control = 288, GDM = 347) were relatively small. Studies incorporating larger numbers of subjects are needed to conclusively evaluate this issue.

## 5. Conclusions

The increased levels and lactonase activities of PON1 in women with GDM compared with the control women suggest that a compensatory stimulation of PON1 lactonase activities is present in women with GDM. Increased oxidative stress, *-108C/T* and/or *192Q/R* variants of the *PON1* gene, and abnormalities in lipoprotein metabolism may be related to these changes. These findings may provide a basis for elucidating the pathogenesis of GDM and potentially preventing and treating the disease.

## Figures and Tables

**Table 1:** Clinical characteristics of the women with GDM and the women with uncomplicated pregnancies.

	Controls (*n* = 288)	GDM (*n* = 347)	*P*
Age (years)	31.38 ± 4.49	33.50 ± 4.40	<0.001
Gestation age (weeks)	39.14 (38.86, 39.43)	39.14 (38.86, 39.57)	0.124
Parity	1 (1, 2)	2 (1, 2)	0.093
Prepregnancy BMI (kg/m^2^)	20.74 ± 2.67	22.21 ± 3.39	<0.001
Weight gain during pregnancy (kg)	14.58 ± 4.29	12.14 ± 4.34	<0.001
Delivery BMI (kg/m^2^)	26.47 ± 2.96	26.91 ± 3.38	0.091
SBP (mmHg)	113.44 ± 10.05	115.47 ± 10.36	0.014
DBP (mmHg)	72.14 ± 8.36	73.20 ± 9.19	0.138
Neonatal birth height (cm)	49.88 ± 1.56	49.64 ± 1.77	0.095
Neonatal birth weight (g)	3393.64 ± 355.00	3375.66 ± 446.53	0.606
Insulin treatment (*n*)	0	49	

Values are presented as mean ± SD or median (lower limit of IQR, upper limit of IQR) unless otherwise noted. BMI: body mass index; SBP: systolic blood pressure; DBP: diastolic blood pressure.

**Table 2:** Metabolic profile, oxidative stress indices, and PON1 activities of the women with GDM and the women with uncomplicated pregnancies.

	Controls (*n* = 288)	GDM (*n* = 347)	*P*	*P* ^a^
Metabolic profile				
Fasting glucose (mmol/L)	4.24 (3.91, 4.60)	4.30 (3.97, 4.86)	0.002	0.003
Fasting insulin (pmol/L)	61.95 (46.53, 95.01)	74.10 (49.03, 118.41)	0.002	0.001
HOMA-IR	1.67 (1.20, 2.80)	2.08 (1.23, 3.74)	0.001	0.001
TG (mmol/L)	3.39 (2.79, 4.37)	3.74 (3.00, 4.78)	0.001	0.031
TC (mmol/L)	6.13 ± 1.21	6.07 ± 1.14	0.531	0.580
HDL-C (mmol/L)	2.04 ± 0.49	2.02 ± 0.49	0.585	0.331
LDL-C (mmol/L)	3.16 ± 1.01	2.99 ± 0.88	0.026	0.062
Non-HDL-C (mmol/L)	4.10 ± 1.10	4.06 ± 0.97	0.604	0.829
AI	2.16 ± 0.72	2.11 ± 0.65	0.598	0.525
TG/HDL-C	1.79 (1.34, 2.27)	2.00 (1.43, 2.66)	0.006	0.023
apoA1 (g/L)	2.24 ± 0.45	2.19 ± 0.34	0.117	0.163
apoB (g/L)	1.22 ± 0.29	1.22 ± 0.27	0.981	0.891
apoB/apoA1	0.56 ± 0.16	0.56 ± 0.14	0.572	0.491

Oxidative stress indices and PON1 activities				
TOS (nmol H_2_O_2_ Equiv./mL)	23.87 ± 8.20	26.96 ± 10.38	0.001	<0.001
TAC (mmol Trolox Equiv./L)	1.09 ± 0.18	1.15 ± 0.17	<0.001	0.001
OSI	21.51 ± 7.85	22.67 ± 8.09	0.156	0.115
MDA (nmol/mL)	5.57 ± 1.59	6.17 ± 1.53	<0.001	<0.001
PON1 lactonase activity (U/mL)	11.39 ± 2.50	11.90 ± 2.46	0.022	0.018
PON1 level (mU/mL)	29.59 ± 6.45	30.70 ± 6.25	0.049	0.062
NLA	386.07 ± 39.89	388.93 ± 35.41	0.383	0.171

Values are presented as mean ± SD or median (lower limit of IQR, upper limit of IQR). HOMA-IR: homeostatic model assessment of insulin resistance; TG: triglycerides; TC: total cholesterol; HDL-C: high-density lipoprotein cholesterol; LDL-C: low-density lipoprotein cholesterol; apo: apolipoprotein; AI: atherosclerosis index; TOS: total oxidant status; TAC: total antioxidant capacity; MDA: malondialdehyde; OSI: oxidative stress index; PON1: paraoxonase 1; NLA: normalized lactonase activity. ^a^All comparisons of the parameters were corrected for differences in age and BMI at delivery.

**Table 3:** Frequencies of PON1 genotype and allele in the women with GDM compared with the women with uncomplicated pregnancies.

		Controls (*n* = 288)	GDM (*n* = 347)	*x* ^2^	*P*
Genotype					
-108	CC	87 (30.2%)	102 (29.4%)		
	CT	134 (46.5%)	171 (49.3%)		
	TT	67 (23.3%)	74 (21.3%)	0.549	0.760
192	QQ	36 (12.5%)	44 (12.7%)		
	QR	120 (41.7%)	158 (45.5%)		
	RR	132 (45.8%)	145 (41.8%)	1.132	0.568

Allele frequency					
-108	C	0.535	0.540		
	T	0.465	0.460	0.040	0.841
192	Q	0.333	0.354		
	R	0.667	0.646	0.622	0.430

Data of genotype are presented as the number (%) of patients or controls.

**Table 4:** Lactonase activity, level, and NLA of PON1 and markers of oxidative stress according to PON1 genetic polymorphisms in the women with GDM and the women with uncomplicated pregnancies.

	PON1 -108C/T			PON1 192Q/R		
	CC	CT	TT	QQ	QR	RR
Controls						
*n*	87	134	67	36	120	132
Age (years)	31.79 ± 4.69	31.66 ± 3.78	30.31 ± 5.33	31.68 ± 4.44	32.06 ± 4.47	30.69 ± 4.44^¶^
Delivery BMI (kg/m^2^)	26.52 ± 2.48	26.39 ± 2.63	26.57 ± 4.02	25.95 ± 2.34	26.60 ± 2.46	26.49 ± 3.50
TOS (nmol H_2_O_2_ Equiv./mL)	23.88 ± 7.90	22.97 ± 8.28	25.78 ± 8.35	25.39 ± 6.76	22.24 ± 8.19	24.76 ± 8.46
TAC (mmol Trolox Equiv./L)	1.11 ± 0.18	1.08 ± 0.19	1.09 ± 0.16	1.16 ± 0.19	1.06 ± 0.17^§^	1.10 ± 0.18
OSI	21.83 ± 7.51	20.94 ± 7.26	22.35 ± 7.26	22.58 ± 7.48	20.67 ± 8.84	21.91 ± 7.02
MDA (nmol/mL)	5.63 ± 1.62	5.56 ± 1.70	5.49 ± 1.30	6.13 ± 1.69	5.23 ± 1.47^§^	5.74 ± 1.63^¶^
PON1 lactonase activity (U/mL)	12.81 ± 2.39	11.14 ± 2.46^†^	10.05 ± 1.71^†, ‡^	12.72 ± 2.23	11.50 ± 2.79^§^	10.93 ± 2.15^§^
PON1 level (mU/mL)	32.74 ± 6.16	29.09 ± 6.32^†^	26.51 ± 5.30^†, ‡^	30.88 ± 5.69	29.58 ± 6.54	29.24 ± 6.58
NLA	392.78 ± 37.62	383.06 ± 40.84	383.38 ± 40.53	413.73 ± 36.54	386.19 ± 40.13^§^	378.46 ± 37.50^§^

GDM						
*n*	102	171	74	44	158	145
Age (years)	33.61 ± 4.46	33.62 ± 4.26	33.08 ± 4.64	33.89 ± 4.48	32.89 ± 4.43	34.03 ± 4.28^¶^
Delivery BMI (kg/m^2^)	26.85 ± 4.24	26.93 ± 2.82	26.95 ± 3.28	26.81 ± 5.31	26.78 ± 2.80	27.08 ± 3.23
TOS (nmol H_2_O_2_ Equiv./mL)	26.84 ± 9.82	26.73 ± 11.11	27.59 ± 9.59	27.77 ± 10.56	26.13 ± 9.97	27.56 ± 10.77
TAC (mmol Trolox Equiv./L)	1.16 ± 0.16	1.13 ± 0.16	1.18 ± 0.17	1.12 ± 0.14	1.16 ± 0.18	1.15 ± 0.16
OSI	22.63 ± 7.87	22.51 ± 8.42	23.06 ± 7.80	24.22 ± 8.92	21.76 ± 7.30	23.18 ± 8.59
MDA (nmol/mL)	6.19 ± 1.43	6.08 ± 1.57	6.33 ± 1.56	6.35 ± 1.42	6.06 ± 1.54	6.23 ± 1.54
PON1 lactonase activity (U/mL)	13.46 ± 2.28	11.54 ± 2.18^†^	10.47 ± 2.11^†,‡^	12.79 ± 1.94	12.28 ± 2.55	11.21 ± 2.34^§,¶^
PON1 level (mU/mL)	34.22 ± 6.02	29.76 ± 5.82^†^	27.75 ± 5.38^†,‡^	31.66 ± 5.25	31.09 ± 6.44	29.92 ± 6.35
NLA	394.94 ± 30.63	389.97 ± 36.23	379.29 ± 38.31^†,‡^	405.35 ± 25.90	395.86 ± 27.61	377.28 ± 41.11^§,¶^

Values are as the means ± SD. TOS: total oxidant status; TAC: total antioxidant capacity; MDA: malondialdehyde; OSI: oxidative stress index; PON1: paraoxonase 1; NLA: normalized lactonase activity. All comparisons of oxidative stress markers and PON1 activities were corrected for differences in age and delivery BMI between the two groups. ^†^*P* < 0.01 compared with the PON1 gene -108 CC genotype subgroup; ^‡^*P* < 0.05 compared with the PON1 gene -108 CT genotype subgroup. ^§^*P* < 0.05 compared with the PON1 gene 192 QQ genotype subgroup; ^¶^*P* < 0.05 compared with the PON1 gene 192 QR genotype subgroup.

**Table 5:** Association of important independent variables with lactonase activity, level, and NLA of PON1 by multivariate regression analysis in the women with and without GDM.

Independents	Unstandardized coefficients		Standardized coefficients	*t*	*P*
	*B*	S.E.M.	*β*		
Model I: PON1 lactonase activity as dependent variable					
Constant	7.879	1.120		7.033	<0.001
PON1 -108C/T^a^	-1.557	0.121	-0.496	-12.819	<0.001
apoA1 (g/L)	2.656	0.278	0.375	9.567	<0.001
apoB (g/L)	1.116	0.333	0.131	3.353	0.001
TAC (mmol Trolox Equiv./L)	1.608	0.577	0.110	2.786	0.006
Age (years)	-0.053	0.020	-0.102	-2.606	0.010

Model II: PON1 level as dependent variable					
Constant	18.867	2.419		7.799	<0.001
PON1 -108C/T^a^	-4.126	0.355	-0.531	-11.617	<0.001
apoA1 (g/L)	6.946	0.729	0.396	9.527	<0.001
PON1 192Q/R^b^	2.346	0.371	0.290	6.363	<0.001
MDA (nmol/mL)	0.484	0.117	0.116	2.738	0.006
Age (years)	-0.141	0.050	-0.110	-2.797	0.005
apoB (g/L)	1.684	0.844	0.080	1.995	0.047

Model III: NLA as dependent variable					
Constant	419.407	11.896		35.255	<0.001
PON1 192Q/R^b^	-25.529	1.856	-0.618	-15.907	<0.001
TAC (mmol Trolox Equiv./L)	31.346	8.507	0.146	3.685	<0.001
MDA (nmol/mL)	-3.759	0.994	-0.153	-3.780	<0.001
apoB (g/L)	14.472	4.979	0.116	2.907	0.004

PON1: paraoxonase 1; apo: apolipoprotein; MDA: malondialdehyde; NLA: normalized lactonase activity; TAC: total antioxidant capacity. ^a^PON1 -108C/T (CC = 1, CT = 2, and TT = 3). ^b^PON1 192Q/R (QQ = 1, QR = 2, and RR = 3).

**Table 6:** Association of lactonase activities, level, and genetic polymorphisms of PON1 with the neonatal birth height and weight by multivariate regression analysis in the women with and without GDM.

Independents	Unstandardized coefficients		Standardized coefficients	*t*	*P*
	*B*	S.E.M.	*β*		
Model I: neonatal birth height as dependent variable					
Constant	50.863	0.344		148.057	<0.001
PON1 levels (mU/mL)	-0.038	0.011	-0.147	-3.404	0.001

Model II: neonatal birth weight as dependent variable					
Constant	3599.794	86.686		41.525	<0.001
PON1 levels (mU/mL)	-7.142	2.804	-0.110	-2.547	0.011

PON1: paraoxonase 1.

## Data Availability

The data used to support the findings of this study are available from the corresponding author upon request.
